# Hepatitis C and *Helicobacter Pylori*

**DOI:** 10.4103/0974-777X.68542

**Published:** 2010

**Authors:** Viroj Wiwanitkit

**Affiliations:** *Wiwanitkit House, Bangkhae, Bangkok, Thailand - 10160*

Sir,

I read with great interest the recent publication by El- Masry *et al*. on Hepatitis C and *Helicobacter pylori*.[1] El- Masry *et al*. concluded that, “It may be stated that our results collectively reflect a remarkable increase in *H. pylori* prevalence with advancing hepatic lesions, and the eradication treatment may prove beneficial in those patients with chronic hepatitis C.”[1] Indeed, both hepatitis C and *Helicobacter pylori* infections are problematic infections in many developing countries. It is not surprising that a copresentation can be seen in some settings. The question that remains unanswered is whether there is any clinical importance of such co-presentations. There are some recent interesting publications on this topic. Recently, a paper reported the possibility of both hepatitis C and *Helicobacter pylori* as being causes of lymphoma.[2] Both infections are also mentioned for their relationship to primary immune thrombocytopenia.[3] For sure, future researches are required for supporting such proposals. I agree with the statement of Mamun-Al-Mahtab that “*Helicobacter pylori* and hepatitis C together hamper health.”[4]
